# The stop-feed effect of cholecalciferol (vitamin D_3_) and the efficacy of brodifacoum combined with cholecalciferol in Y139C-resistant Norway rats (*Rattus norvegicus*)

**DOI:** 10.1007/s10340-023-01600-0

**Published:** 2023-02-23

**Authors:** Nicole Klemann, Bernd Walther, Franz-Rainer Matuschka, Jens Jacob, Stefan Endepols

**Affiliations:** 1 Klemann Consult, Federal Research Centre for Cultivated Plants, 48231 Warendorf, Germany; 2 Julius Kühn Institute (JKI), Institute for Plant Protection in Horticulture and Forests and Institute for Epidemiology and Pathogen Diagnostics, Rodent Research, Federal Research Centre for Cultivated Plants, Toppheideweg 88, 48161 Münster, Germany; 3 Universität Potsdam, Hochschulambulanz, 14469 Potsdam, Germany; 4 Envu, Solution Development EMEA, 40789 Monheim, Germany

**Keywords:** Norway rat, Anticoagulant resistance, Cholecalciferol, Vitamin D_3_, Brodifacoum, Rodent management

## Abstract

Second-generation anticoagulant rodenticides potentially build persistent residues in animals and accordingly pose a risk of secondary poisoning. We examined the effect of a low concentration of cholecalciferol in brodifacoum bait on bait consumption by Norway rats (*Rattus norvegicus* Berkenhout 1769) and on the control success in a laboratory study and in field trials. Additionally, the efficacy of both baits was determined against resistant Y139C rats. Cholecalciferol caused a strong stop-feed effect after two days in the laboratory study. On two field study sites each, bait containing either 25 mg kg^−1^ brodifacoum or 25 mg kg^−1^ brodifacoum and 100 mg kg^−1^ cholecalciferol was applied to treat infestations of Norway rats. Infestations were assessed pre- and post-treatment. Rats were radio-tagged, and carcasses were searched for during the treatment period. DNA of each rat was genotyped to determine the resistance status conferred by the VKORC1 gene. On all farms, control success exceeded 90%. On farms treated with brodifacoum only, the ratio of total bait consumption to pre-treatment census was significantly higher (6.6 and 4.8 times) than on farms treated with the combination (2.7 and 2.9 times). 78.8% of 183 rats were confirmed Y139C resistant. Bait ingestion was reduced by almost fifty per-cent when cholecalciferol was added to the bait with no impact on control success. All treatments resulted in control levels exceeding 90%, despite a high proportion of anticoagulant-resistant rats. When the use of highly toxic compounds is required in resistance management, addition of cholecalciferol to these baits may reduce the transfer of residues to the environment.

## Introduction

The Norway rat (*Rattus norvegicus*) is known as a commensal rodent pest, coping with a wide range of environmental conditions. Rats are common in rural habitats, in particular on livestock farms, where rat colonies settle and individuals actively disperse over long distances (Herden 1992; Taylor and Quy 1978; Taylor 1978; Telle 1966). Rats and house mice (*Mus musculus*) contribute to the dissemination of pathogens into livestock by building an epidemiological bridge between wild animals and livestock and between different herds of livestock (Backhans and Fellstrom 2012). Pathogens can be disseminated through excrements, secretions, and carcasses or even physical contact. For example, *Leptospira icterohaemorrhagiae*, the agent of Weil’s disease in humans, is a well-known example of bacteria that are transmitted via urine (Thiermann 1981). *Listeria monocytogenes*, *Campylobacter jejuni*, and the foot-and-mouth disease virus can be shed in feces (Capel-Edwards 1970; Epoke and Coker 1991; Iida et al. 1998). Besides disinfection, rodent management is a necessary biosafety practice for interrupting chains of infection, and regulatory measures on hygiene are stipulated accordingly in livestock farming.

Rodent control largely relies on the use of anticoagulant rodenticides when chemical control is required. Virtually no chemical alternatives exist besides anticoagulant rodenticides for the control of Norway rats, with the exception of cholecalciferol, which was recently authorized in the European Union (EU 2019) and elsewhere. Warfarin-resistant strains of the Norway rat resist not only warfarin, but also some additional anticoagulants, including substances of the second generation. In such areas of resistance, only the most potent anticoagulant rodenticides of the second generation remain a viable choice for efficiently controlling these strains of the Norway rat (RRAC 2021).

Norway rats on livestock farms in Westphalia, Germany, have been a focus of research regarding anticoagulant resistance, when problems controlling them became evident in the early 1970s (Pelz et al. 1995; Telle 1972). Anticoagulant resistance in the Westphalia strain of the Norway rat is linked to a gene on chromosome 1 (Kohn and Pelz 1999). A single nucleotide polymorphism (SNP) in the gene which encodes for the enzyme vitamin K-epoxide reductase (VKORC1) is responsible for the replacement of the amino acid tyrosine by cysteine at position 139 of the enzyme (Rost et al. 2004). The VKORC1 variant tyrosine139cysteine (Y139C) appears to be the sole marker for anticoagulant resistance in Norway rats in Westphalia, because only this SNP has been detected in resistant rats from this area. Laboratory and field trials confirmed that rats and mice of the Y139C strain are not only resistant to anticoagulants of the first generation, but also to the second-generation anticoagulant rodenticides (SGARs) bromadiolone and difenacoum (Buckle et al. 2013; Endepols et al. 2012). Such high level of resistance has not only been determined in Y139C strains, but also in a number of other rats and mouse strains (Baxter et al. 2022; McGee et al. 2020). Consequently, only three SGARs, brodifacoum, flocoumafen, and difethialone, are recommended for use against rats and house mice in such foci of resistance (RRAC 2021).

SGARs potentially build long-lasting residues in animals consuming baits containing such compounds, and therefore, the three most potent ones are considered bio-accumulative (Fisher et al. 2003). Such residues can accumulate in individuals of non-target species when predators or scavengers consume animals or carcasses that have consumed bait. The hazard and risk of secondary poisoning has been a matter of research in recent decades (e.g., Eason and Spurr 1995; Gray et al. 1994; Lund and Rasmussen 1986; Mendenhall and Pank 1980; Smith and Shore 2015).

SGARs were developed to overcome warfarin resistance, and they are generally highly toxic to resistant rats, as well. Bait containing brodifacoum at 10 mg kg^−1^ caused complete kills in resistant rats within only a single day of exposure (Redfern et al. 1976). Commercially available SGAR baits contain the active compound at 25 mg kg^−1^ or at 50 mg kg^−1^. Rats and mice consume anticoagulant baits for several days until symptoms of poisoning occur (Cox and Smith 1992). It was repeatedly documented in laboratory experiments that rats and mice consume multiples of lethal doses of brodifacoum (Frankova et al. 2019; Wheeler et al. 2019). We therefore suppose that they may ingest multiple lethal doses also during practical rodent control, when it relies on the most potent SGARs and good baiting practices, required to achieve complete control.

A method to limit bait ingestion by rats to ideally one dose, instead of multiple lethal doses, may substantially mitigate environmental risks connected to SGAR residues. Cholecalciferol, a pre-hormone known as vitamin D_3,_ is essential for numerous biochemical processes in mammals. It recently attracted attention due to its anti-viral properties and regulating function in the immune system, e.g., in the prevention and treatment of COVID-19 (Brenner et al. 2020; Kitson and Roberts 2012, Castillo et al. 2020), although it is predominantly known for its role in calcium metabolism. In addition to its crucial physiological and immunological functions, the compound is also known for its rodenticide properties, because toxic doses cause hypercalcemia, eventually leading to death some days after ingestion of a lethal dose. Rodenticide products containing 750 mg kg^−1^ of this compound recently have been authorized in Europe (EU 2019) and elsewhere.

During the evaluation of calciferol’s rodenticide properties, a behavioral effect potentially hampering the administration of lethal doses of the baits became evident, a stop-feed effect (Prescott et al. 1992). Administered even in sub-lethal doses, rodents significantly reduce feed intake one to two days after starting to consume calciferol-containing baits. Therefore, the content of cholecalciferol in the bait was adjusted to a high level (Meehan 1984), and baiting campaigns must be diligently prepared and conducted when applying this rodenticide. The question arises whether this stop-feed effect of low cholecalciferol doses may be utilized to limit SGAR-bait ingestion, reducing bait intake to a level not far beyond the lethal dose. We aimed to answer this question, first by a laboratory feeding study with cholecalciferol, then by a field study comparing bait consumption and control success when bait contained either brodifacoum or brodifacoum and cholecalciferol.

## Materials and methods

### Laboratory study

A choice-feeding study was conducted to test the stop-feed effect induced by calciferol-containing bait in the absence of an anticoagulant rodenticide. Three groups (control group, Group A, and Group B) of ten rats (*Rattus norvegicus*) each were subjected to the experiment. The control group and Group A consisted of male rats; Group B consisted of female rats. All animals were healthy adults, weighing at least 230 g. We did not expect any impact of sex on consumption of un-treated diet in the control group. The rats were laboratory-bred descendants of a strain that had been derived from wild-trapped, warfarin-susceptible brown rats from four locations in Germany. Each group was kept in three connected pens (20 cm × 60 cm) with boxes for shelter, saw-dust, water, and standard laboratory diet (standard rat diet; Höveler, EQUOVIS GmbH, Münster Germany) under standard laboratory conditions. The rats acclimatized in the test setup for ten days, with standard laboratory diet ad libitum. No serious aggressive behavior was observed during the acclimatization period or during bait exposure.

The test-bait formulation was a paste bait, based on vegetable fat and cereal flour, and contained cholecalciferol at 100 mg kg^−1^. The test bait was provided by Bayer AG Crop Science-R&D, Monheim, Germany. During the choice-feeding trial, the rats of both treatment groups a and b received the cholecalciferol-containing bait and the challenge diet (standard rat diet; Höveler, Germany) ad libitum. The control group received the same bait as the treatment groups but without cholecalciferol, and the challenge diet. Consumption of bait and challenge diet was recorded daily for 14 days in all groups. The challenge diet was ground, and the paste bait was thoroughly pressed into the feed containers to avoid translocation. Pens were carefully inspected daily for remains of challenge diet and bait. To compare bait consumption among groups, the daily consumption was normalized by its relation to the bodyweight of the respective group.

### Field study

The field study was conducted on four farms with livestock farming in North Rhine-Westphalia, Germany. The farms were at least 2.3 km apart. Farmers stated that they had not used rodenticides on their properties for at least six months prior to the study. Based on careful site surveys, rat infestations were supposed to be at about the same level on all farms. Structure and habitat characteristics were similar on all farms, and particularly, farms #1 and #2, as well as farms #3 and #4 were very similar in their extension, in particular, number and distance between buildings. The farm numbers were allocated to the experimental sites in the sequence of first visiting them. Farms #1 and #2 were treated at the same time, and farms #3 and #4 were simultaneously treated two months later.

Farms #1 and #3 were treated with paste bait, containing brodifacoum at 25 mg kg^−1^. Farms #2 and #4 were treated with paste bait with the same inerts, but containing brodifacoum 25 mg kg^−1^ and cholecalciferol 100 mg kg^−1^. Both bait samples were provided by Bayer AG, Crop Science, R&D, Monheim, Germany. Rolled oats for the feeding census were purchased locally at the agro-dealer RCG Warendorf.

The field trials were carried out according to the principles of the ECHA guide on the evaluation of rodenticides (ECHA 2017) with the following schedule: Implementation of the trial, trapping and radio-tagging, pre-treatment census for three days, followed by two days break, treatment period for 32–35 days, followed by two days break, post-treatment census for three days.

Assessments of the infestations were based on the presence of excrements, damage, and footprints. A large number of census feed points were installed in order to assess the infestation size in and around buildings on the farms (Table 1). Whenever possible, positions of census feed points were not the same as the position of treatment bait points (at least 1 m distance). Locations of bait points (numbers per site see Table 1) were chosen according to the interactive rodent control program Bay Tool (Endepols et al. 2003). Bait was offered in plastic bait stations (Rattenköderbox “B,” Detia Garda GmbH, Germany).

**Table 1:** Number of census points and treatment bait points, and number of rats radio-tagged on each of the four study sites

Farm ID	Bait (a.i.)	Treatment period; Days	Census points	Tracking plates	Treatment bait points	Radio-tagged rats
#1	BR	32	41	18	27	14
#2	BRCC	32	30	11	19	15
#3	BR	35	14	8	14	15
#4	BRCC	35	19	10	13	14

*a.i.* Active ingredient, *BR* brodifacoum, *BRCC* brodifacoum and cholecalciferol

Two indirect census methods were employed, both prior to and after the treatments. For feeding census, 100–200 g rolled oats, exceeding the assumed daily take by the rodents, were filled in bait stations. The census feed points were checked daily for three days. The number of feed sites (census feed points with take) and the amount of the census feed taken, measured to the nearest gram, were recorded daily. All census feed was removed on the last day of the census assay. The total amount of census feed consumed provided an assessment of the population size.

For a second independent census, the rats’ activity was assessed using tracking plates (patches of ca. 20 cm × 20 cm silver sand). Tracking activity was measured for three days, simultaneously with the feeding census (Table 1). The locations of the patches were mapped and inspected for signs of activity and resurfaced daily. A robust scoring system was devised to assess the number of rodent footprints per patch in relation to the percentage coverage of the tracking plates’ surface with tracks in five classes: 0% (index 0), 1–5% (index 1), 5–33% (index 2), 34–66% (index 3), and > 66% (index 4). The daily tracking activity was given as the sum of the index values on all patches recorded after 24 h. All tracking records were taken by the same person to avoid subjective bias. By observing the fate of radio-tagged rats, the above indirect census methods were validated.

On each farm, between 18 and 30 live traps of various types (Schwengber, Kortenbrede GmbH, and Tomahawk live trap) were set at suitable locations inside and outside buildings where rat activity was detected. Rat trapping was conducted for 6–10 days on each farm, and traps were checked at least every 12 h. Traps were equipped with rolled oats and pieces of non-woven dust sheet for nesting.

Trapped rats were transferred to a veterinary anesthesia workstation to slightly anesthetize the animals by inhalation of an isoflurane-oxygen mix (2.5–5%). Sedated rats were weighed to the nearest gram. Rats weighing > 200 g were equipped with a radio collar (TXE-116CZ, Telenax, Mexico) that emitted a signal at an individual frequency changing in pulse rate when the internal movement sensor did not detect movement for four to five hours. Radio-tagged rats were released after full recovery at the point of capture.

During the course of the study, the position of each radio-tagged rat was determined daily with a three-element yagi antenna (Linflex, Biotrack Ltd, UK) and a VHF receiver (Australis 26 k, Titley Scientific, Australia) until the radio signal indicated death of an animal, which was immediately recovered if possible. Additional carcasses found in proximity of dead radio-tagged animals or during daily searches for carcasses were also collected. All carcasses were stored at − 22 °C.

### Statistical analysis of infestation sizes and of control success

To compare the initial infestation sizes on the four study sites, the pre-baiting census values on each farm (in gram/24 h per feed point) were analyzed for differences by performing rank-sum tests (Mann–Whitney, U-test). All census points with takes during the last day of the pre-treatment census (i.e., points with consumption) were included in the analysis.

Population censuses before and after treatments were compared to quantify the success of the tested product samples in controlling Norway rats for each infestation. The degree of control was expressed as percentage reduction based on the values of pre-baiting treatment consumption.

Survival rate was defined as follows: Survival rate (%) = (gram census feed post-treatment census × 100)/gram census feed pre-treatment census.

To run the rank-sum tests (Mann–Whitney, U-test) for comparing the post-baiting census results, the ratio of post-baiting census consumption values of each feed point to the pre-baiting value of the entire respective study site was calculated, based on the 24 h consumption during the last day of each census. The ratio of total bait consumption during the treatment period to the total consumption on Day 2 of the pre-treatment census was calculated to provide a normalized estimate of the total consumption per rodent during the treatment period. Finally, treatment success as calculated on the census data for each study site was related to the proportion of radio-tagged rats found dead.

### Genetic analysis of the VKORC1 gene for Y139C

DNA of a tissue sample from the tail of dead rats mostly collected during or after the treatment was isolated using the DNeasy Blood and Tissue Kit (Qiagen, Hilden, Germany) and included in a high-resolution melting polymerase chain reaction (HRM-PCR), which amplifies a 141 bp fragment of the Exon 3 of the vkorc1 gene, using the PikoReal Real-Time PCR system (Biozym Scientific GmbH, Hessisch Oldendorf, Germany) (Diaz and Kohn 2020). Comparing the melting curves, variations at position 139 are detected and assigned as wildtype, heterozygote, or homozygote. In addition, randomly selected amplification products were sequenced to confirm the assignment using a 4300 DNA Analyzer (LI-COR Biosciences, Lincoln, NE, USA [36].

## Results

### Laboratory study

**Fig. 1:**
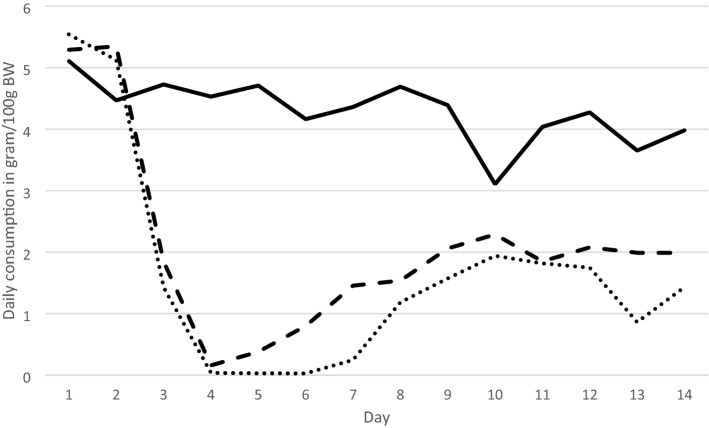
Daily consumption of bait (gram per 100 g bodyweight (BW) in three groups of Norway rats (*n* = 10 each), given a choice between challenge diet (data not shown) and bait with or without cholecalciferol (100 mg kg^−1^). The control group (males, solid line) was provided with challenge diet and bait without cholecalciferol. Group A (males, dotted line) and Group B (females, dashed line) were offered a choice of challenge diet and bait containing 100 mg kg^−1^ cholecalciferol

Both, control and treatment groups accepted the baits well on the first day and continued to consume at an almost equal level on Day 2. All groups preferred the bait over the challenge diet, exceeding 90% of total consumption on the first day in all groups. The lowest bait takes were observed from Day 3 to Day 7 in the treatment groups a and b, which reduced their total daily consumption of bait to almost zero by Day 4. During the second week, the treatment groups slowly resumed consumption by Day 7 (Fig. 1). After 7 days, Group A had consumed 481 g bait and 62 g challenge diet, Group B 322 g versus 93 g, and the control group 1389 g versus 79 g, respectively. Daily Mean consumption per 100 g bodyweight was 4.55 g (SD = 1.71 g) in Group A, 3.64 g (SD = 1.52 g) in Group B, and 7.49 g (SD = 0.50 g) in the control group. The stop-feed effect was evident on Day 3, when the treatment groups consumed 50 g and 62 g of cholecalciferol bait, while the control group consumed 208 g of the bait not containing cholecalciferol (Fig. 1).

### Field study

**Fig. 2:**
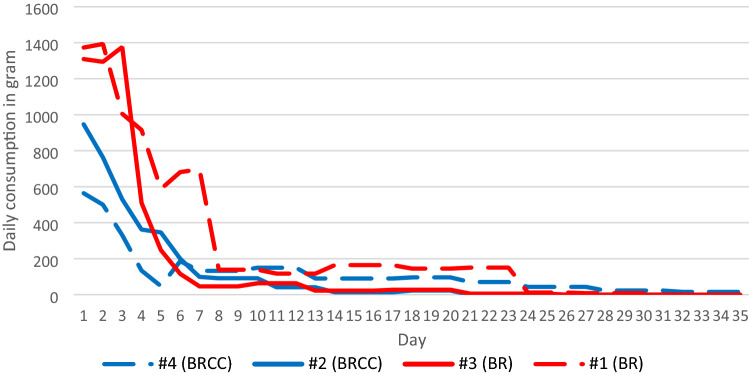
Bait consumption by Norway rats in field trials where brodifacoum (BR, red) or brodifacoum and cholecalciferol (BRCC, blue) was used

The initial rat infestations were similarly sized based on pre-treatment consumption censuses ranging from 1,138 g/24 h to 1,416 g/24 h (Table 2, Fig. 2). No significant difference was found for pre-treatment values comparing the four study sites by pairwise rank-sum test (*p* > 0.05, U-Test, Table 3). Mean daily consumption per active pre-census point was in the range of 74.6 g to 103.5 g.

**Table 2:** Summary of field trial data on four study sites treated with brodifacoum (BR) or brodifacoum and cholecalciferol (BRCC)

Farm ID:	#1 (BR)	#2 (BRCC)	#3 (BR)	#4 (BRCC)
Pre-treatment census (g/24 h)	1368	1416	1138	1342
Rats found dead	38	26	26	21
Total bait consumption (g)	9032	3768	5411	3887
Total bait consumption/24 h pre-treatment census	6.6	2.7	4.8	2.9
Control success by feed-census (%)	99	98	100	92
Control success by tracking score (%)	97	94	100	95
Survivors/radio-tagged	1/14	2/15	1/15	2/14

The fourth row gives the ratio of total bait consumption and consumption during 24 h pre-treatment as a normalized estimate of the total bait consumption per rodent during the treatment period. Control success as calculated by two census methods, and mortality of radio-tagged rats on four study sites treated with brodifacoum (BR) or brodifacoum and cholecalciferol (BRCC)

*a.i* active ingredient, *BR* brodifacoum, *BRCC* brodifacoum + cholecalciferol

**Table 3:** Pre-baiting census in four study sites

Farm ID (a.i.)	N	Median take (gram)	Mean take per census point (gram)	#2 (BRCC)	#3 (BR)	#4 (BRCC)
#1 (BR)	11	65	103.5	81.5 (*P* = 0.692)	81.5 (P = 0.445)	80.5 (*P* = 0.962)
#2 (BRCC)	18	38	76.2	–	140.5 (*P* = 0.588)	160.0 (*P* = 0.779)
#3 (BR)	17	34	83.3		–	144.0 (*P* = 0.418)
#4 (BRCC)	18	47	74.6			–

Number of census points with takes during pre-baiting census (*N*), median daily consumption during pre-baiting census, and U-values and P (in brackets) of rank-sum tests (Mann–Whitney) comparing the pre-baiting census pairwise among study sites

*a.i.* active ingredient, *BR* brodifacoum, *BRCC* brodifacoum and cholecalciferol

On all farms, control success exceeded 90% within 32–35 treatment days (Table 2, Fig. 2), independent of the type of bait used. There was no statistically significant difference in the control level values between the sites (*p* > 0.05, U-Test, Table 4). Median consumption per post-treatment feed point was 0 g on each experimental site.

**Table 4:** Post-baiting census in four study sites

Farm ID (a.i.)	*N*	Median take (gram)	Mean take per census point (gram)	#2 (BRCC)	#3 (BR)	#4 (BRCC)
#1 (BR)	11	0	0.0	82.5 (*P* = 0.828)	82.5 (*P* = 0.174)	71.5 (*P* = 0.317)
#2 (BRCC)	18	0	0.8	–	148.5 (*P* = 0.269)	138.5 (*P* = 0.238)
#3 (BR)	17	0	1.6		–	127.5 (*P* = 0.065)
#4 (BRCC)	18	0	7.1			–

Number of census points with takes during pre-baiting census (N), median in daily post-baiting census, and U-values and P (in brackets) after rank-sum test (Mann–Whitney) comparing post-baiting census values (individual post-baiting census point value/total pre-baiting census value of study site per 24 h) between sites

*a.i.* active ingredient, *BR* brodifacoum, *BRCC* brodifacoum and cholecalciferol

Total bait consumption varied between 3768 and 9032 g. On the two farms treated with BR, the ratio of total bait consumption to pre-treatment census for a period of 24 h was 6.6 and 4.8. On farms treated with BRCC, this indicator of quantitative bait consumption was 2.7 and 2.9 (Table 2). One or two radio-tagged rats survived the treatments on each site. Between 21 and 38 rats per study site were found dead during the treatment.

Tissue samples from the tail of 183 carcasses were successfully analyzed by HRM-PCR for the resistance-mediating polymorphism Y139C (Table 5). Additionally, 81 of these samples were randomly selected for sequencing, and the results of the HRM-PCR were confirmed. On all study sites, rats carrying the resistance marker Y139C were present. The mean proportion of resistant rats was 78.8% (47.7–97.2%). 17.1 to 100% of the resistant rats were heterozygous, and 0 to 82.9% were homozygous for Y139C (Table 5).

**Table 5:** Resistance status on four study sites, frequency of the Y139C polymorphism in samples of Norway rat

Farm ID (a.i.)	N	% susceptible	% resistant	Resistant rats	
				% homozygous	% heterozygous
#1 (BR)	58	12.1	87.9	54.9	45.1
#2 (BRCC)	45	17.8	82.2	24.3	75.7
#3 (BR)	44	52.3	47.7	0.0	100.0
#4 (BRCC)	36	2.8	97.2	82.9	17.1
Total	183	21.2	78.8	40.5	59.5

*a.i.* active ingredient, *BR* brodifacoum, *BRCC* brodifacoum and cholecalciferol

## Discussion

Cholecalciferol contained in bait at 100 mg kg^−1^ induced a strong stop-feed effect in Norway rats starting within 48 h in a laboratory feeding study. No effect on palatability was observed, as the rats readily consumed the bait during the first day of exposure. The content of cholecalciferol in the tested bait was not considered to be lethal, as the oral LD_50_ of cholecalciferol is 30–100 mg kg^−1^ (Meehan 1984). Commercial baits with cholecalciferol as the only compound therefore contain 750–1000 mg kg^−1^. With such a concentration, rats ingest a lethal dose before the stop-feed effect acts under real-life conditions. The effect of calciferols on bait ingestion has been examined (Greaves et al. 1974; Prescott et al. 1992), although referring to bait containing 750 mg kg^−1^. Two effects were described that result in reduced bait intake, a physiological effect soon after dosing, and a conditioned taste aversion, called bait shyness, after recovery from sub-lethal dosing (Prescott et al. 1992). The stop-feed effect appeared after 48 h in our laboratory feeding study with 100 mg kg^−1^, similar to an ingestion of bait with 750 mg kg^−1^ (Prescott et al. 1992). Rats, which consumed bait during the first hours of access, may have expressed the effect on the first day already. We conclude, therefore, that the effect appears to be time-dependent rather than dose-dependent, if a, yet unknown, minimum dose has been ingested. There was a slight recovery of bait consumption after four days. It remains hypothetical whether this behavior was a kind of habituation, or whether it was caused by declining serum levels of hydroxy-cholecalciferol after highly elevated levels caused by the first 48 h consumption.

Brodifacoum, similar to other highly potent SGARs, has been proven effective against Norway rats and house mice, including all warfarin-resistant strains identified by SNPs on the VKORC1 gene (Greaves et al. 1988; McGee et al. 2020; RRAC 2021). The efficacy of brodifacoum in controlling Y139C resistant rats has already been confirmed in field trials in Westphalia (Buckle et al. 2012). This is reflected in our results for rat infestations with a high frequency of Y139C resistance, where > 90% control success was reached independent of the presence of cholecalciferol in the bait. This supports recommending this compound for resistance management.

The efficacy of all treatments has been assessed using three methods of census. Each method has its pros and cons. Therefore, we used this combination of well acknowledged direct and indirect census methods (Backhans and Fellstrom 2012). The feeding census, as employed here, is the most essential indirect method in assessing the efficacy of rodenticides against commensal rodents (ECHA 2017). The less accurate tracking census confirmed the feeding census very well in this study. In addition to these methods, we determined the mortality of radio-tagged rats, a method considered as a direct longitudinal evaluation (Cowan and Townsend 2015). Only one or two radio-tagged rats survived the treatments, although the previous procedure of trapping and tagging may have deterred these rats from entering any rodent control device, such as bait stations. This observation can be considered a strong proof that a feeding census, as conducted here, is a reliable method for assessing rodent control measures.

Controlling two rat infestations with bait containing brodifacoum at 25 mg kg^−1^ resulted in almost 100% control success of the rat infestations on Farm 1 and 3, respectively. As common with this type of field study, the complete rat-free status can rarely be achieved, and the feeding census always bares a minor level of uncertainty, due to roaming and migrating rats, immigrating other small mammal species, alternative food, and individual feeding behavior of rats (Cowan and Townsend 2015; Quy et al. 1992). One radio-tagged rat survived on each farm. Conclusively, control success was not 100%, but close to it. Such high levels of control exceeding 90% are required for product authorization according to international guidelines (ECHA 2017).

Rats and mice consume anticoagulant baits for several days until symptoms of poisoning occur. We calculated the ratio of total bait consumption during the treatment period to the total consumption on Day 2 of the pre-treatment census on each study site to compare the bait consumption between treatments. The ratio of total brodifacoum bait consumption during the treatment period to the consumption on Day 2 of the pre-treatment census was calculated to be 6.6 and 4.8 for farm 1 and 3, respectively. Using bait, containing the combination of brodifacoum at 25 mg kg^−1^ and cholecalciferol at 100 mg kg^−1^ resulted in similarly high (98% and 92%) control success rates of the rat infestations on Farm 2 and 4. This high success rate was achieved with a ratio of total bait consumption to the pre-treatment census being only 2.7 and 2.9. This represents a 59.1% (first pair of study sites) and 39.6% (second pair) reduction in the normalized bait consumption in rats treated with the combination compared to those treated with the bait containing only brodifacoum.

Thus, by combining brodifacoum with a sub-lethal dose of cholecalciferol, rats still consumed a lethal dose of brodifacoum, but their bait intake was curtailed much more rapidly than without cholecalciferol. The high mortality despite the clear reduction in bait take also indicates that the extra 50% brodifacoum consumed by the rodents on Farm 1 and 3 was not required for management efficacy. Thus, it posed an unnecessary potential deposition of the rodenticide into the environment and associated risks of secondary poisoning (Eason and Spurr 1995; Mendenhall and Pank 1980).

However, the raw proportion of the two experimental baits consumed gives only an estimate of the initial emission of the anticoagulant compound. The proportion of ingested bait and residues in the body and in organs, such as the liver, depends on numerous factors. Depending on the progress of digesting the bait and metabolic elimination of the anticoagulant, the relation to the quantity of bait ingested might be vague. The metabolism of anticoagulant residues in the bodies of mammals is biphasic. After consumption of excessive doses of a persistent compound, such as brodifacoum, there is a rapid initial elimination of excess and unbound active substance (Horak et al. 2018). Additionally, second-generation anticoagulant rodenticides, such as brodifacoum, are excreted largely unchanged in feces (Horak et al. 2018).

Initial metabolism is rapid; often a large percentage of the compound is cleared within the first days after ingestion. The second phase of elimination is a slow metabolic depletion of the bound portion of the residue that is much smaller than the portion excreted in the first phase (Horak et al. 2018). Thus, part of the excess active substance may be excreted or eliminated before the death of the animal due to anticoagulant poisoning, or before the animal is killed and consumed by a predator. A rat may fall prey immediately after consuming bait. In such a case and when consumed completely, the exposure of the predator will closely relate to the amount of bait consumed by its prey. In contrast, when consuming a moribund rat after it had stopped feeding for days, the predator’s exposure will be lower. The level of residues of SGARs in whole body carcasses largely depends on the amount of bait consumed and time period between bait consumption by the rodent and the consumption of the rodent by a predator. The longer this period is, the weaker the correlation of residues and bait consumption should be. Future research is required to model the exposure of predators in relation to the temporal patterns of bait consumption by commensal rodents under field conditions.

The addition of cholecalciferol to highly toxic bait may reduce the environmental impact of residues of compounds required in resistance management where a considerable probability cannot be prevented of predators and scavengers exposed to poisoned rodents. With the present study, it was proven that the addition of cholecalciferol results in decreased consumption of an anticoagulant with no substantial negative impact on the efficacy in one of the most resistant rat strains. The effect of decreased consumption has potential positive impact not only in resistant, but also in susceptible rodent infestations, and possibly on SGAR residues in target rodents. Further research is required with other SGARs to examine how the stop feeding effect impacts on bait consumption, in particular when resistant strains of rats and mice shall be controlled.

## Author contributions

SE, NK, and JJ planned the field studies. SE and F-RM steered the laboratory work. NK, BW, and SE conducted the field work. SE wrote the first draft; all authors contributed to and approved the final manuscript.

## Data Availability

Not applicable.
